# Oxidation and Reduction Dual-Responsive Polymeric Prodrug Micelles Co-delivery Precisely Prescribed Paclitaxel and Honokiol for Laryngeal Carcinoma Combination Therapy

**DOI:** 10.3389/fphar.2022.934632

**Published:** 2022-07-22

**Authors:** Lanzhu Zhou, Jun Wu, Zhe Sun, Wenzhong Wang

**Affiliations:** Department of Otorhinolaryngology Head and Neck Surgery, The First Affiliated Hospital of Bengbu Medical College, Bengbu, China

**Keywords:** honokiol, paclitaxel, polymeric prodrug, dextran, diselenium bond, stimuli-responsive, glutathione, reactive oxygen species

## Abstract

Laryngeal carcinoma is the most common head and neck malignancy globally, and chemotherapy is still the most common treatment for this type of carcinoma. Monotherapy has become powerless because of the lack of drugs in the anticancer agent library, the difficult process of new drug discovery, and the widespread drug resistance. Combination therapy with two agents, in particular Chinese herbal medicines with chemotherapy drugs, is a potential alternative to chemotherapy alone. However, combination therapy faces difficulties in delivering multiple drugs to tumor tissue in a precise ratio. Here, a cocktail polymeric prodrug micelle (PHPPM) was developed using an oxidation and reduction dual-responsive polymeric paclitaxel (PTX) and polymeric honokiol (HK) prodrugs. Both of them were obtained by covalently conjugating the drug to dextran *via* diselenium bonds. Following optimization and characterization, the PHPPM with the precise mass ratio of PTX and HK was obtained, enabling ratiometric drug loading, synchronized drug release in response to tumor high-level reactive oxygen species and glutathione environment, long blood circulation, and high tumor accumulation. This co-delivery system can effectively inhibit laryngeal carcinoma growth *in vitro* and *in vivo*. Codelivery of chemotherapy agents and Chinese herbal medicine with a precise ratio and controlled release of the two drugs at the tumor site provides an effective approach to clinical therapy for other laryngeal carcinomas.

## Introduction

Laryngeal carcinoma is the most common head and neck malignancy globally (Kumar et al., 2018). In 2020, over 180,000 new cases of laryngeal carcinoma were diagnosed, with approximately 100,000 new deaths (Sung et al., 2021). Surgery, chemotherapy, and radiotherapy are commonly used to treat laryngeal carcinoma in clinical practice today (Wu et al., 2020). Among them, chemotherapy remains one of the most common strategies for laryngeal treatment because of its superior efficacy (Bonomi et al., 2018). However, the efficacy of monotherapy with a single drug was greatly hindered by toxic side effects and drug resistance (Meng et al., 2021). Through the synergistic effect of two drugs, combination chemotherapy has been shown to significantly improve anticancer efficacy and reduce side effects when two or more therapeutic drugs with different acting sites and mechanisms of anticancer activity are used simultaneously.

In recent years, attention has been increasingly paid to combining Chinese herbal medicines with chemotherapy drugs that can result in a synergistic response that may eliminate the tumor while reducing side effects (Fu et al., 2018; Dasari et al., 2022). Honokiol (HK), a traditional Chinese medicine isolated from *Magnolia Officinalis*, showed anticancer, antidepressant, antioxidant, and anti-inflammatory effects (Lin et al., 2007; Dikalov et al., 2008; Xu et al., 2008). Studies have shown that HK can inhibit the growth of laryngeal, liver, colon, lung, breast, and brain cancer (Banik et al., 2019). Moreover, HK has been shown to significantly improve the treatment effects of traditional chemotherapy drugs, such as paclitaxel (PTX) and doxorubicin (Wang et al., 2017; Zou et al., 2018). Moreover, combining HK with traditional chemotherapy agents may be an effective strategy for treating laryngeal carcinoma. However, the poor water solubility of both PTX and HK made it difficult to use these two drugs together in clinical practice. Moreover, because of the different pharmacokinetics and biodistribution of the individual drugs, co-delivery of different drugs to tumor sites at the precise drug dosages is difficult to accomplish, resulting in unmatched dosage combinations that make it difficult to achieve efficient synergistic therapy or even cause antagonism (Li et al., 2020). In addition, the overlapping toxicity of different agents with co-administration also poses a challenge to combination chemotherapy.

Nanotechnology-based drug delivery systems have helped solve some of the problems mentioned above. Nano formulations can effectively increase solubility, tumor targeting, pharmacokinetics, and biodistribution, as well as reduce the side effects of chemotherapeutic agents (Cai et al., 2020; Zeinali et al., 2020; Wang D et al., 2021). Nano-drug delivery systems, in particular, can simultaneously deliver multiple drugs to the same focal point at a predetermined drug ratio (Zhang et al., 2020). Various nano-drug delivery systems for cancer combination therapy have been developed to date. Many have been applied in clinical trials, such as Trilimus, a nano-drug delivery system prepared by encapsulating paclitaxel, 17-N-allylamino-17-demethoxygeldanamycin (17-AAG), and rapamycin in poly (ethylene glycol)-block-poly (D,L-lactic acid) (PEG-*b*-PLA) formed micelles (Zhang et al., 2016).

Polymeric prodrug micelles (PPM) are prepared by covalently linking drugs to biocompatible polymers with smart bonds to form amphiphilic polymers that can then form core-shell micelles in an aqueous solution. They have gathered a lot of attention in the current nanomedicine world (Su et al., 2018; Zhang et al., 2019; Hu et al., 2020). Thus, PPM possesses the advantages of polymer micelles and prodrug strategies, such as high drug-loading capability, significantly reduced premature agent release in the blood circulation, and good bionic characteristics (Huang et al., 2018; Ibrahim et al., 2019; Wang Y et al., 2021). The PPM can control drug release in response to tumor microenvironment, such as high glutathione (GSH), high reactive oxygen species (ROS), and low pH, when the drugs are linked by a diselenide bond, disulfide bond, or ester bond (Tabatabaei Rezaei et al., 2016; Taresco et al., 2018; Zhuang et al., 2018; Sui et al., 2019; Zuo et al., 2020; Fu et al., 2021 ). Among these stimuli, GSH has been demonstrated to be abundant in the cytoplasm of cancer cells (∼10 mM) but absent in the extracellular environment (2–20 µM) (Tu et al., 2021). In addition, it has been reported that the ROS level in cancer cells was 1,000-fold higher than in normal tissue (Mollazadeh et al., 2021). Thus, GSH and ROS constitute two appropriate triggers for drug release.

**FIGURE 1:**
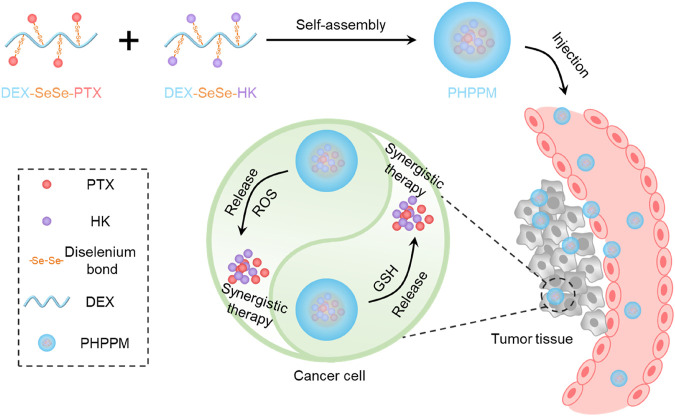
Schematic illustration of the fabrication of paclitaxel and honokiol co-loaded cocktail polymeric prodrug micelles (PPM), and intracellular glutathione/reactive oxygen species (ROS) dual-responsive drug release for precise combination therapy.

Dextran (DEX), a natural polysaccharide, has the advantages of facile functionalization, excellent water solubility, low toxicity, biodegradability, and good biocompatibility (Chen et al., 2020). Moreover, the use of DEX as a drug carrier is more stable and adsorb fewer nonspecific proteins than PEG (Li et al., 2017). These advantages indicate that DEX is an ideal candidate for designing and preparing DEX-based drug delivery systems, especially PPM. Hence, we designed and prepared a GSH/ROS dual-responsive cocktail PPM (PHPPM) co-delivery paclitaxel and honokiol for the laryngeal carcinoma combination therapy (Figure 1). The PHPPM comprises of a precise ratio of GSH/ROS-responsive PTX (PTX-SeSe-DEX) and HK (HK-SeSe-DEX) polymeric prodrugs. The PTX-SeSe-DEX and HK-SeSe-DEX were fabricated by conjugating PTX or HK to DEX through diselenide bonding. The diselenide bond can be cleavable by intracellular over-produced ROS and GSH to release PTX and HK, which may kill cancer with a synergistic effect after being internalized by cancer cells.

## Materials and Methods

The details of chemicals and reagents, instruments, cell culture, and animals are provided in supporting information.

### Synthesis of SeSe-PTX and SeSe-HK

According to a previous report, diacid anhydride of 3,3′-diselenodipropionicacid (DSPA) was prepared before synthesizing of SeSe-PTX and SeSe-HK (Sun et al., 2019). In brief, 10 mmol of DSPA was mixed with 10 ml of acetic anhydride and stirred at 30°C under the protection of nitrogen gas (N_2_). The mixture was mixed with 15 ml of methylbenzene after a 2 h reaction, and then the solvent was removed under reduced pressure. After three repetitions, a crude anhydride product was obtained, which was immediately dissolved in 50 ml of anhydrous dichloromethane. Afterwards, PTX (10 mmol) and 4-dimethylaminopyridine (DMAP, 1 mmol) were added to the mixture. The reaction was maintained at 30°C in an N_2_ atmosphere for 2 h. The mixture was dried under reduced pressure, and the product obtained was purified by silica gel column chromatography with methyl alcohol: dichloromethane (1:20) to obtain SeSe-PTX (yield: 76.7%). The same preparation method was used to obtain SeSe-HK (yield: 68.2%). The products were measured by ^1^H NMR.

### Synthesis of Dextran-SeSe-PTX and Dextran-SeSe-HK

*N,N′*-carbonyldiimidazole (CDI) catalysis synthesized DEX-SeSe-PTX and DEX-SeSe-HK. To prepare DEX-SeSe-PTX, SeSe-PTX (3 mmol) was reacted with CDI (4 mmol) in 30 ml of anhydrous dichloromethane under N_2_ protection at 30°C for 4 h. The solvent was then removed under reduced pressure to yield CDI-SeSe-PTX. Thereafter, CDI-SeSe-PTX was mixed with DEX (0.3 mmol) in formamide. The mixture was vigorously stirred at room temperature for 48 h under N_2_ protection. After the reaction was completed, the mixture was extensively dialyzed (molecular weight cut-off (MWCO): 3.5 kDa) in 70% (v/v) methyl alcohol for 48 h and distilled water for 48 h. To obtain DEX-SeSe-PTX, the solution was lyophilized and stored at −20°C in an N_2_ atmosphere for further use. Moreover, DEX-SeSe-HK was also obtained using the same protocol. ^1^H NMR was used to confirm the structure of drug conjugations.

The drug conjugation rate (DCR) was measured using the high-performance liquid chromatography (HPLC) method and calculated using the following equation:

DCR( %)= Mass of drug/Mass of polymeric prodrug×100%.

### Polymeric Prodrug Micelle Preparation

The dialysis method was used to prepare PPM. In brief, 20 mg of DEX-SeSe-PTX and 10 mg of DEX-SeSe-HK were dissolved in 5 ml of dimethyl sulfoxide (DMSO) using sonication, followed by dialysis (MWCO 3.5 kDa) against deionized water at 4°C for 16 h in the dark. The solution was then filtered through a 0.45 μm membrane and lyophilized to get redox-sensitive **PTX** and **HK** co-loaded **PPM**, denoted as **PHPPM**. The same method was used to prepare **PPM** formed by DEX-SeSe-**PTX** (named PPPM) and PPM formed by DEX-SeSe-HK (**HPPM**).

In summary, the HPLC method measured the drug loading capability (DLC) of PTX and HK. To calculate the DLC, the following formula was used:

DLC(wt.%)=Mass of Drug in PPM/Mass of PPM×100%.

Moreover, coumarin-6 loaded PPMs were prepared. In brief, 20 mg of DEX-SeSe-PTX, 10 mg of DEX-SeSe-HK, and 0.5 mg of coumarin-6 were dissolved in 5 ml of DMSO, and the solution was dialyzed (MWCO 3.5 kDa) against deionized water at 4°C for 16 h in the dark. The solution was then filtered through a 0.45 μm membrane and lyophilized to obtain PHPPM loaded with coumarin-6. Likewise, the same method was used to make coumarin-6 loaded PPPM and HPPM.

### Stability Evaluation

Fresh prepared PPMs were dispersed in either phosphate-buffered saline (PBS, pH 7.4) with or without 10% of fetal bovine serum (FBS). Furthermore, the cocktail PPM solution was cultured at 37°C with some shaking. Dynamic light scattering (DLS) measured the size of PPMs at predetermined time points (0, 4, 8, 12, 24, 36, or 48 h).

### *In vitro* Drug Release

The PHPPM drug release was evaluated by the classical dialysis method. The release medium was PBS (pH 7.4) buffer with 1% (m/v) of Tween80 and various concentrations of GSH (0, 1, and 10 mM) or hydrogen peroxide (H_2_O_2_) (0, 1, and 10 mM).

The PHPPM (including 2.0 mg of PTX and 1.0 mg of HK) was dissolved in 1 ml of PBS before being placed in a dialysis bag (MWCO 3.5 kDa). The dialysis bag was immediately immersed in 99 ml of release media and maintained at 37°C with some shaking. At pre-set time intervals, 2 ml of release medium outside the bag was withdrawn, and 2 ml of fresh release media was added. The released amount of PTX and HK was detected using the HPLC method presented in the supporting information.

### Cellular Uptake

Approximately 1 × 10^4^ of Hep-2 cells were seeded into a well of a six-well plate and incubated overnight when cell growth was approximately 70–80%. Thereafter, cells were incubated with coumarin-6 loaded PHPPM, PPPM, or HPPM with 500 ng/ml of coumarin-6. After being incubated for 1 or 4 h, the cells were washed three times with cold PBS, fixed with 4% formaldehyde, stained with 4′,6-diamidino-2-phenylindole, and observed under a fluorescence microscope (Leica DMI6000B, Germany).

### Cytotoxicity

Hep-2 cells were seeded at a density of 5 × 10^3^ cells per well in 96-well plates. After a 24 h culture period, the medium was replaced with 150 μl of free drug or PPMs dispersed medium and incubated for another 48 h. Thereafter, 20 μl of MTT solution was added to each well and cultured for another 4 h. Moreover, the medium was replaced with 100 μl of DMSO to dissolve the formazan. In conclusion, the absorbance of each well was measured using a BioTek microplate reader.

### Pharmacokinetics and Biodistribution

SD rats were used as an animal model to test the pharmacokinetics. In general, SD rats were given a single intravenous injection of free PTX, free HK, and PHPPM at the drug concentration of 10 mg/kg. At pre-set time points, 200 µl of blood was collected from the orbit of each mouse. The plasma was then obtained by centrifuging the blood samples at 3,000 rpm at 4°C for 10 min. After that, 200 µl of acetonitrile and 200 µl of dichloromethane were added to an equal plasma volume to extract the PTX or HK. After centrifugation, the supernatant was collected, concentrated, re-dissolved in methanol, and subjected to HPLC to measure the free drug or conjugation drug.

Male BALB/c mice were used as animal models in the biodistribution study, with subcutaneous human laryngeal carcinoma Hep-2 cells tumor xenografts injected at 1 × 10^6^ cells. The mice were randomly divided into three groups (*n* = 3) when the tumor volume reached approximately 500 mm^3^ and treated with free PTX, HK, or 2/1PHPPM at a 10 mg/kg drug concentration. Mice were sacrificed after being treated for 12 h. The major organs, including the heart, liver, spleen, lung, kidneys, and tumor, were collected, washed in PBS, and weighed. In summary, the tissues were homogenized in PBS, and the free drug or drug conjugation was extracted with acetonitrile and dichloromethane, and analyzed using HPLC.

### Hemolysis Analysis

Fresh blood obtained from healthy SD rats was stored in heparinized tubes. Furthermore, 1 ml of fresh blood was centrifugated at 1,200 rpm for 3 min to separate the red blood cells, then re-dispersed in PBS. Then, 50 µl of re-dispersed red blood cells were added to PPMs solutions at different drug concentrations (0.5, 1, 2.5, 5, 10, 15, or 20 μg/ml). Under the same conditions, red blood cells were mixed with distilled water and PBS (pH 7.4) as positive and negative controls, respectively. Each sample was incubated at 37°C for 4 h after gently shaking, and the absorbance of the supernatant was measured using a BioTek microplate reader at a wavelength of 540 nm. The hemolysis rate was calculated using the following formula:

$$\text{Hemolysis rate}=\left({\text{OD}}_{S}{-\text{OD}}_{n}\right)\text{/}\left({\text{OD}}_{p}{-\text{OD}}_{n}\right)\times 100\text{\%}.$$

OD_*s*_, OD_*n*_, and OD_*p*_ are the absorbance (optical density, OD) of the sample, PBS, and water groups, respectively.

### *In vivo* Antitumor Evaluation

The Hep-2 tumor-bearing mice model was created by injecting 150 μl of Hep-2 cell suspension (2 × 10^6^ cells per mice) into the right armpit of the mice. When the tumor volume reached 50–80 mm^3^, mice were randomly divided into eight groups, five mice each. Moreover, the mice were treated with PBS, PTX, HK, PTX + HK, PHPPM, PPPM, or HPPM at a dose of 6 mg/kg of PTX or 3 mg/kg of HK. These drug formulations were injected into the tail vein on days 0, 3, and 6. The width and length of the tumor and the mouse weight were recorded every 3 days. The following formula was used to calculate tumor volume:

$$\text{Volume }\left({\text{mm}}^{3}\right)\;=1\text{/}2\left({\text{Length × Width}}^{2}\right).$$

On day 15, the tumor tissues were harvested, washed, and weighed. The tumor suppression rate (TSR) based on the weight of the extracted tumor was calculated using the following equation:

TSR(%)= (1 -Tumor weight of the test group/Tumor weight of the PBS group)×100%.

### Statistical Analysis

The student’s *t*-test was used to analyze the significant differences between the two groups using origin software 2021b. *p* < 0.05 was considered statistically significant.

## Results and Discussion

### Polymeric Prodrug Synthesis and Characterization

**FIGURE 2:**
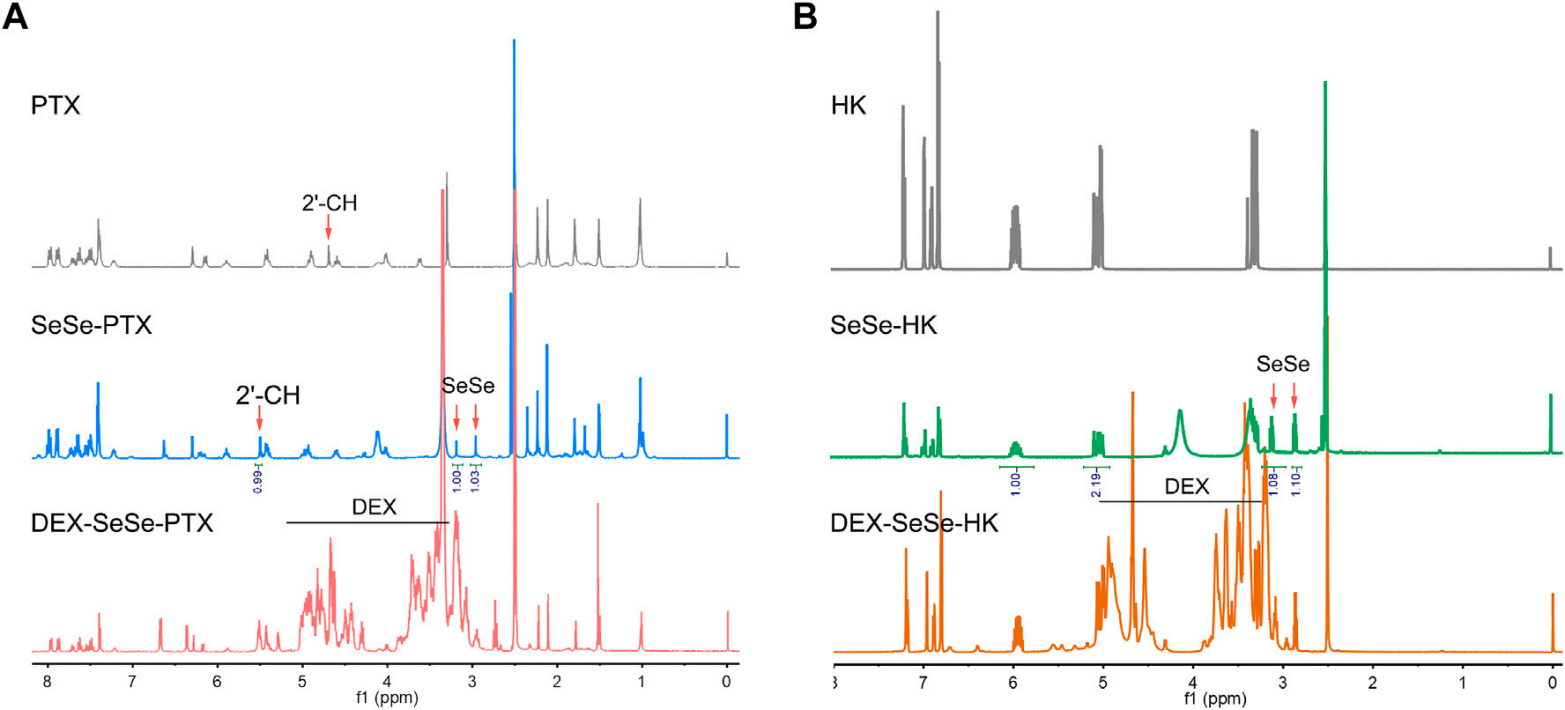
Characterization of polymeric prodrugs. **(A–B)** ^1^H nuclear magnetic resonance spectrums of parent drugs and polymeric prodrugs in dimethyl sulfoxide-_*d6*_: **(A)** paclitaxel (PTX), SeSe-PTX, and DEX-SeSe-PTX spectrums; **(B)** honokiol (HK), SeSe-HK, and DEX-SeSe-HK spectrums.

The redox-sensitive polymeric prodrugs DEX-SeSe-PTX and DEX-SeSe-HK were fabricated through a two-step esterification reaction, and the synthetic routes are presented in Supplementary Figure S1. Diacid anhydride of 3,3′-diselenodipropionicacid (DSPA) was prepared and used instead of DSPA because of the high reactivity of anhydride with hydroxyl groups (Bao et al., 2014). As a result, the formation of the byproduct could be drastically reduced. PTX or HK was reacted with the diacid anhydride of DSPA to obtain the SeSe-PTX and SeSe-HK, respectively. The products were confirmed using ^1^H NMR. As shown in Figure 2A, the chemical shifts of DSPA were assigned in the range of *δ*2.5–3.0 ppm in the spectrums of SeSe-PTX and SeSe-HK, indicating that these two prodrugs were successfully prepared. In comparison to the PTX spectrum, the 2ʹ-CH proton peak in SeSe-PTX was 5.6 ppm, whereas it was 4.7 ppm in the PTX spectrum, suggesting that the esterification reaction between DSPA and PTX occurred at the 2ʹ-hydroxyl of PTX.

Furthermore, SeSe-PTX and SeSe-HK were conjugated to DEX to obtain DEX-SeSe-PTX and DEX-SeSe-HK. ^1^H NMR and UV spectrum confirmed that the conjugation was successful. As presented in Figure 2B, the signals of the benzene ring of PTX and HK could be observed in the spectrums of DEX-SeSe-PTX and DEX-SeSe-HK, respectively, demonstrating that both prodrugs were successfully synthesized. The DCR of DEX-SeSe-PTX, and DEX-SeSe-HK were determined to be (4.78 ± 0.38) % and (7.58 ± 0.43) %, respectively, using HPLC by a standard curve method (Supplementary Figure S2).

The critical micelle concentration (CMC) of DEX-SeSe-PTX and DEX-SeSe-HK was 21.2 μg/ml and 26.9 μg/ml, respectively, using the Nile red as the fluorescence probe (Supplementary Figure S3). The low CMC value suggests that both prodrugs that formed PPMs might have good dilution stability in the blood circulation.

### Polymeric Prodrug Micelles Preparation and Characterization

**FIGURE 3:**
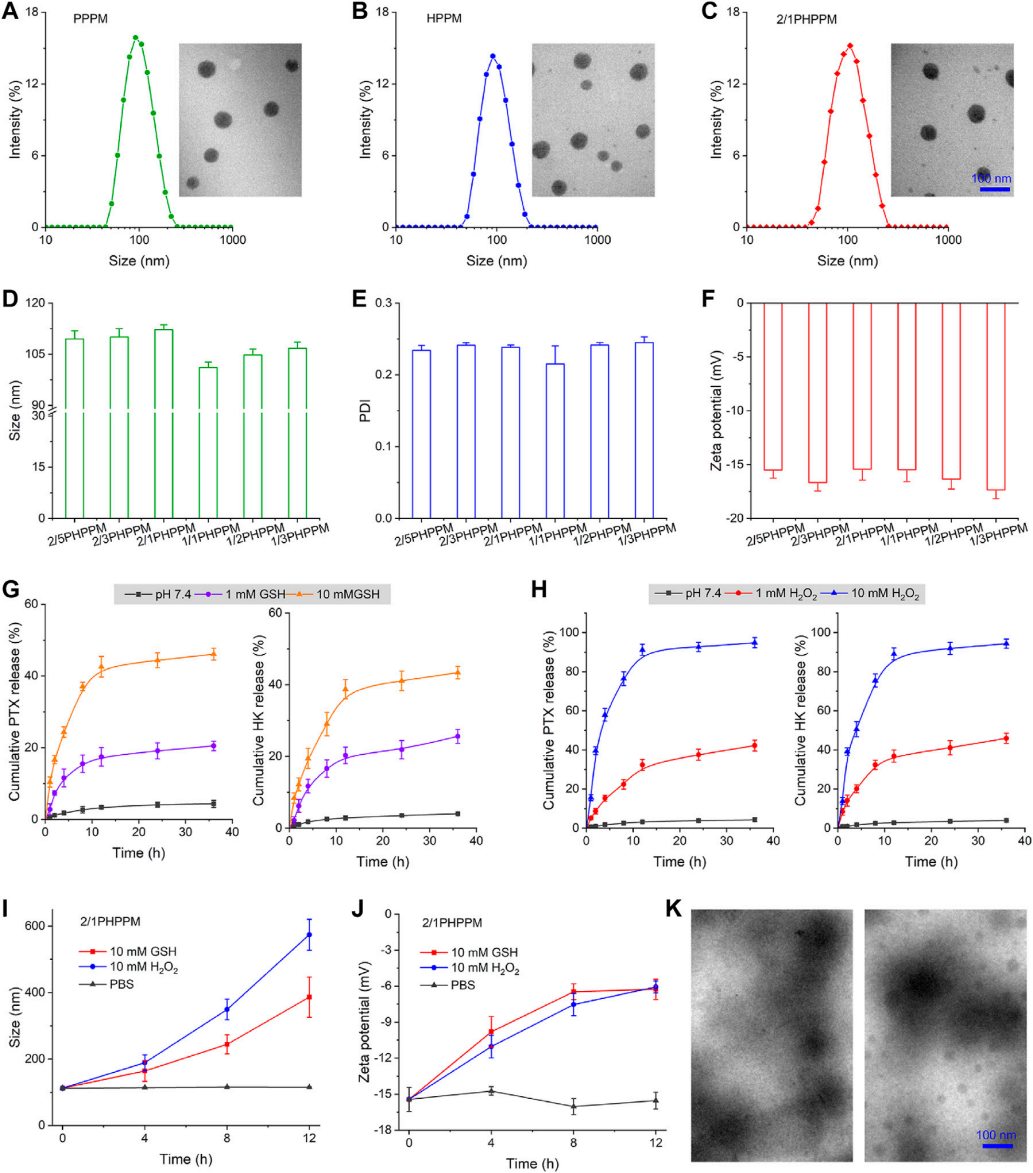
Preparation and characterization of PPMs. **(A–C)** the hydrodynamic particle size and transmission electron microscopy (TEM) images of PPPM **(A)**, HPPM **(B)**, and 2/1PHPPM **(C)**. **(D–F)** Size **(D)**, polydispersity index **(E)**, and zeta potential **(F)** of PHPPMs with different polymeric paclitaxel (PTX) and honokiol (HK) ratios. **(G–H)** the cumulative release of PTX and HK from 2/1PHPPM in the presence of different concentrations of glutathione (GSH) **(G)** or hydrogen peroxide (H_2_O_2_) **(H)**. **(I–J)** the changes in size **(I)** and zeta potential **(J)** of 2/1HPPPM following incubation with 10 mM GSH or H_2_O_2_ for 0, 4, 8, and 12 h. **(K)** transmission electron microscope images of 2/1HPPPM following incubation with 10 mM GSH or H_2_O_2_ for 12 h. Data are represented as the mean ± standard deviation, *n* = 3.

PPMs with similar nano-properties and precisely controlled drug loading are critical for combination therapy because of the customizable prescription and predictable *in vivo* fate. The amphiphilicity of DEX was conferred by conjugating hydrophobic PTX or HK to its side, allowing these two polymeric prodrugs to self-assemble into PPMs in water solution. The two-drug-loaded cocktail PPM and single-drug-loaded PPM were produced by the simple dialysis method to investigate the influence of single- or double-drug loading on the physicochemical properties of the yielded PPMs. The single **PTX**- or **H**K-loaded **PPMs** were denoted as **PPPM** and **HPPM**, respectively, while the double-drug-loaded cocktail PPM was denoted as **PHPPM.** As shown in Figures 3A,B, both PPPM and HPPM had a similar size of approximately 100 nm, a narrow polydispersity index (PDI, approximately 0.25), and a spherical shape.

Moreover, different mass ratios of PTX and HK were used to achieve drug loading, demonstrating the ability of independently tunable drug ratios. The mass ratios of PTX and HK, set at 2/5, 2/3, 2/1, 1/1, 1/2, and 1/3 (Supplementary Table S1), were used to denote the cocktails PPMs. Hydrodynamic particle size, size distribution, and zeta potential characterize these PPMs. As presented in Figures 3C–F, all of the PHPPMs had a moderate size (100–120 nm) with a narrow distribution (0.20–0.25) and a moderate zeta potential (−15 to −20 mV). The presented PHPPM transmission electron microscopy (TEM) images of 2/1PHPPM also showed that the PHPPMs were spherical and roughly uniform in size (Figure 3C). Moreover, the stability of PPMs was evaluated by monitoring changes in particle size of PPMs over time in PBS or PBS with 10% FBS at 37°C. As shown in Supplementary Figure S4, the HPPM, PPPM, and 2/1PHPPM were stable under normal conditions, evidenced by no obvious changes in particle size for all PPMs during the 48 h incubation period. Furthermore, the hemolysis rate of PPPM, HPPM, and PHPPM was <5% at drug concentrations ranging from 0.5 μg/ml to 20 μg/ml (Supplementary Figure S5), suggesting that these PPMs were biocompatible (Tsai, 2019; Singh et al., 2020; Tong et al., 2020).

### Redox-Triggered Drug Release

Selenium, an essential trace element for humans, exhibit remarkable protective or inhibitory effects against various cancers, including head and neck neoplasm, breast cancer, and colon cancer (Li and Xu, 2020; Liu et al., 2020). Selenium-containing compounds have unique bond energy, which gives C–Se or Se–Se covalent bonds a dynamic character and makes them responsive to mild stimuli, like GSH and H_2_O_2_, because of the specific electronegativity and atomic radius of selenium (Wei et al., 2020).

The *in vitro* drug release behavior of 2/1PHPPM was investigated using the dialysis method to prove this process. As illustrated in Figure 3G, in the presence of GSH, an improved and sustained drug release behavior of 2/1PHPPM was observed. When 2/1PHPPM was incubated with 1 mM GSH for 36 h, approximately 20 and 25% of PTX and HK were released. Increasing the GSH concentration to 10 mM with the same time scale elicits approximately 46% of PTX and 43% of HK release.

Moreover, the diselenium bond-based polymeric prodrug cocktail was also shown in H_2_O_2_-sensitive drug release (Figure 3H). Selenium bonds may oxidize to form selenone or hydrophilic selenoxide groups after incubation with H_2_O_2_, accelerating the hydrolysis of adjacent ester bonds and releasing the drug from the polymer prodrugs (Wei et al., 2020). When HKPPM was exposed to 10 mM H_2_O_2_, it displayed a rapid drug release behavior. At 36 h, the cumulative release of PTX and HK was approximately 95 and 94%, respectively.

In particular, the release of PTX and HK was almost non-existent in blank media, demonstrating a good stability of the cocktail PPM. The excellent stability of the cocktail PPM reveals that it will not prematurely release its payloads during blood circulation. At the same time, its responsiveness to GSH and H_2_O_2_ bestows the ability rapidly and selectively releases a large amount of PTX and HK inside cancer cells, ultimately killing them.

Using GSH and ROS to remove the hydrophobic drug could transform the hydrophobic core of PPMs into hydrophilic one, resulting in the disassembly of PPMs. DLS and TEM measured the changes in size, zeta potential, and morphology of 2/1HPPPM to confirm this phenomenon. The hydrodynamic particle size of 2/1HPPPM rapidly increased from 100 nm to over 400 nm following incubation with 10 mM GSH or 10 mM H_2_O_2_, as shown in Figure 3I, indicating the disassembly of 2/1HPPPM. Moreover, after being treated with GSH or H_2_O_2_, the zeta potential of 2/1HPPPM was reduced (Figure 3J). Furthermore, TEM results showed that when GSH and H_2_O_2_ were added to 2/1HPPPM, they disintegrated quickly (Figure 3K).

### Cellular Uptake Investigation

**FIGURE 4:**
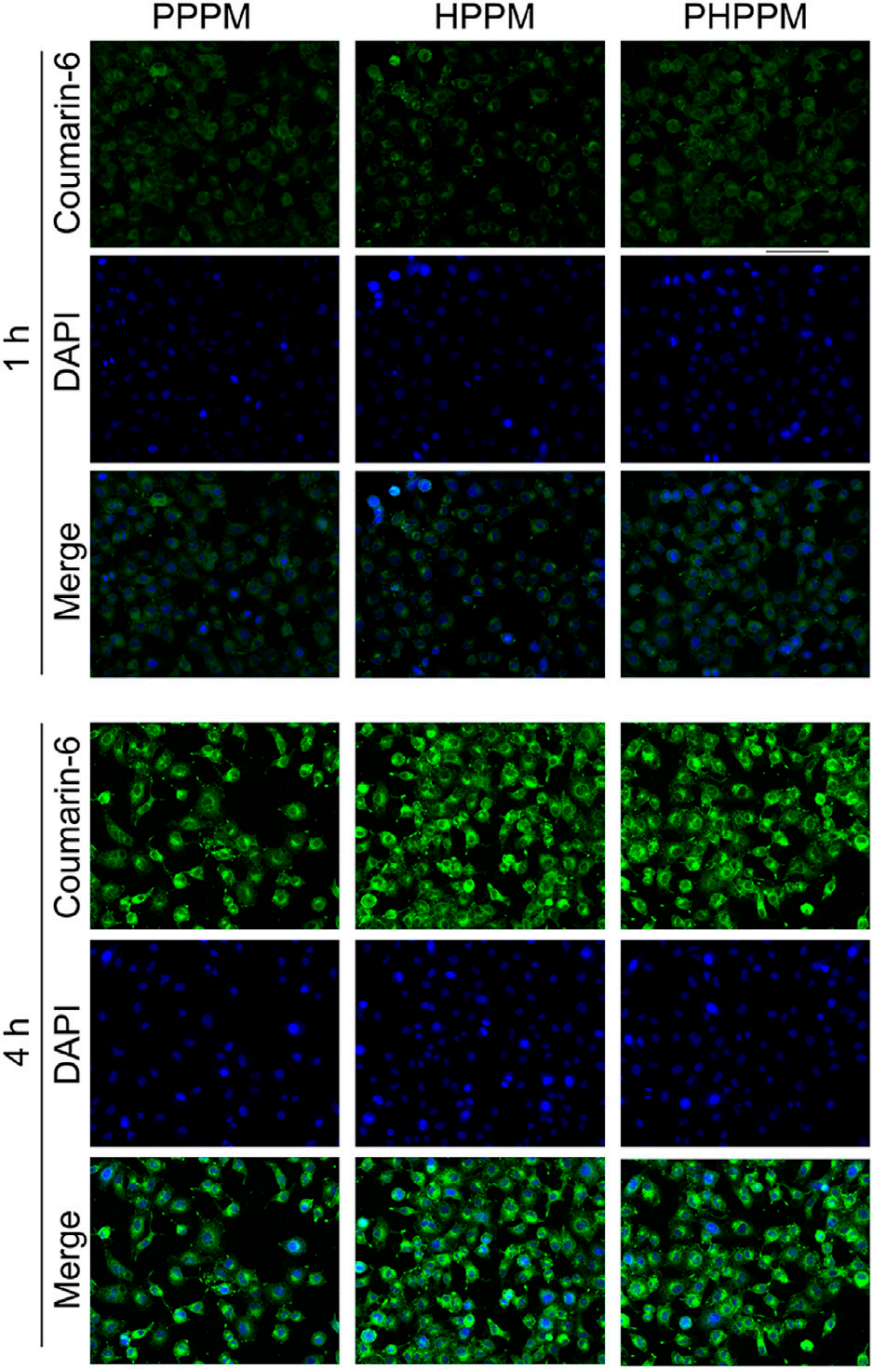
Fluorescence images of Hep-2 cells after 1 or 4 h of treatment with coumarin-6 labeled PHPPM, HPPM, and PPPM.

PPMs internalized by human laryngeal carcinoma Hep-2 cells were observed using a fluorescence microscope. Coumarin-6 was used as the fluorescence probe. Figure 4 shows the green fluorescence of coumarin-6 that can be observed in the cytoplasm after 1 h of incubation with PPMs. In contrast, whereas a 4 h assay showed a higher fluorescence intensity in the cells, the time-dependent fluorescence intensity increases in cells demonstrated that the three PPMs could be effectively internalized by cells. Because of their similar surface properties and particle sizes, the fluorescence intensity in 2/1PHPPM, HPPM, and PPPM treated cells is not significantly different, suggesting that these PPMs exhibit a similar cellular uptake.

### Screening for the Optimal Drug Combinations and Application in Polymeric Prodrug Micelle

**FIGURE 5:**
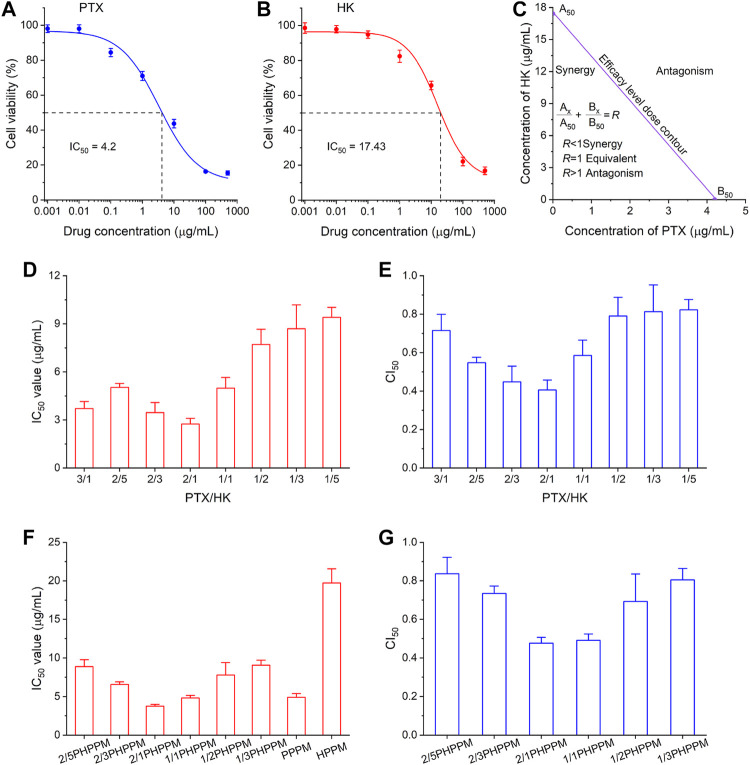
Combination dose screening. **(A–B)** inhibition of different concentrations of polymeric paclitaxel (PTX) **(A)** and honokiol (HK) **(B)** on Hep-2 cells after 48 h. IC_50_ (50% of maximal inhibition) of PTX and HK were 4.2 μg/ml and 17.4 μg/ml, respectively. **(C)** pharmacodynamical synergy and antagonistic PTX and honokiol HK regions. **(D–E)** the IC_50_ **(D)** and combination index_50_ (CI_50_) **(E)** of different ratios of PTX and HK in the synergy area against Hep-2 cells. **(F–G)** the IC_50_ **(F)** and CI_50_ **(G)** cocktails of PPM against Hep-2 cells with different drug loading ratios of PTX and HK.

The current study aimed to co-deliver PTX and HK to tumor cells to effectively kill cancer cells. The most important factor in achieving this goal is determining the best drug-to-drug ratio for combination therapy. The synergistic cytotoxicity of PTX and HK was tested against Hep-2 cells to find the best drug-to-drug ratio for anticancer efficacy. The combination index (CI) was used to measure the combined effect of drugs, including their antagonistic (CI > 1), synergistic (*n* < 1), and additive effect (CI = 1) (Chou, 2018). Chou and Talalay’s formula was used to calculate the CI: CI_50_ = A1/A1_50_ + B1/B1_50_ (Chou, 2010; Banerjee et al., 2021). where A1 and B1 are the 50% inhibitory concentration (IC_50_) values of drug A and drug B, respectively; and A1_x_ and B1_x_ are drug A and drug B in the combination group at the IC_50_ value. The MTT method was used to determine the IC_50_ value of PTX and HK against Hep-2 cells after 48 h. As shown in Figures 5A,B, the IC_50_ values of PTX and HK against Hep cells were 4.2 and 17.4 μg/ml, respectively. The efficacy level dose contour of PTX and HK was plotted to measure the synergistic region of drugs based on their IC_50_ values (Figure 5C). The cytotoxicity of eight fixed-dose combinations of PTX and HK against Hep-2 cells was measured using the MTT method to screen for the optimal molar ratio of PTX and HK. The combinatory molar ratio of PTX and HK was 2:1, and the combination of PTX and HK exhibited a remarkable inhibition effect against Hep-2 cells, with the lowest IC_50_ and CI_50_ values (Figures 5D,E).

Hep-2 cells were treated with a series of cocktail PPMs prepared as described above, and the surviving cells were measured using the MTT method to check whether the 2:1 mass ratios of PTX and HK in the free drug could be applied to PPMs. Similar to free drug combinations, 2/1PHPPMs with a loaded drug mass ratio (PTX/HK) of 2:1 showed the highest cytotoxicity (lowest IC_50_ value) and the best synergistic effect (lowest CI_50_ value) (Figures 5F,G), suggesting that the optimal drug combinations dose for cocktail PPMs can be easily screened using free drugs. Thus, the 2/1PHPPM was used in the following experiments. While the synergistic effect was conserved in both the free drug combination group and the prepared PHPPM group, only the latter can ensure simultaneous delivery of the two drugs to cancer cells in a precise ratio *in vitro* and *in vivo*.

### Pharmacokinetics and Biodistribution

**FIGURE 6:**
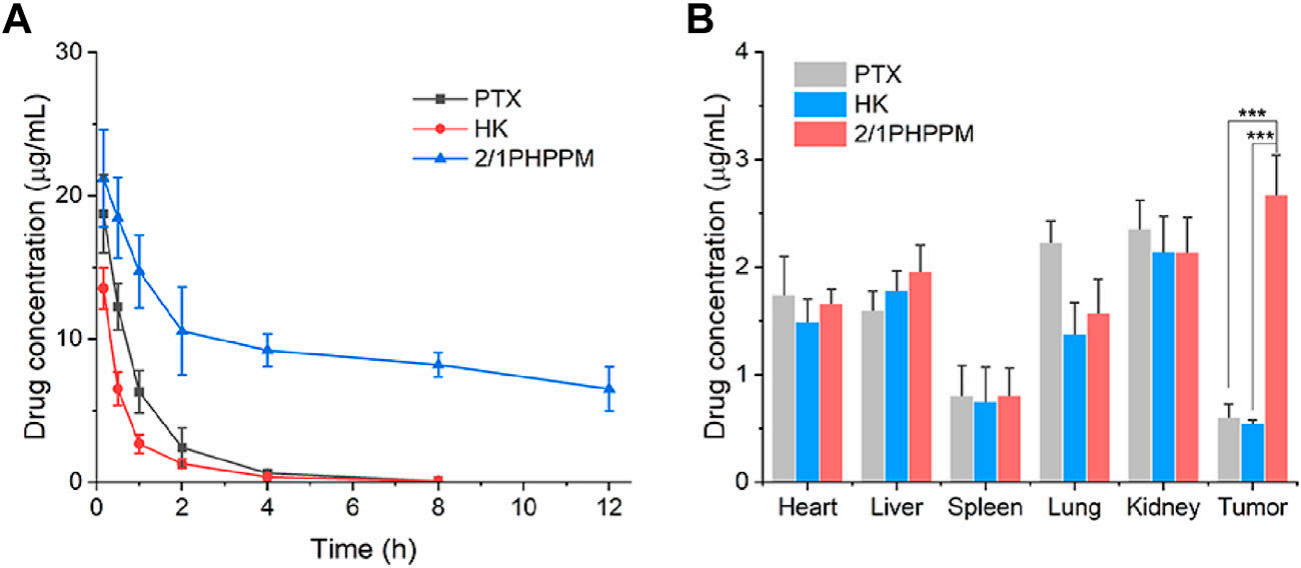
Blood circulation **(A)** and *in vivo* biodistribution **(B)** of paclitaxel, honokiol, and 2/1PHPPM. Data are represented as mean ± standard deviation, *n* = 3. ***p < 0.001.

The blood circulation and tissue distribution of 2/1PHPPM were measured. First, the pharmacokinetic profiles of a free PTX, HK, and 2/1PHPPM were performed in SD rats following intravenous administration to investigate the blood circulation of different drug formulations. As shown in Figure 6A, both free drugs are rapidly cleared from the blood, with the concentrations of PTX and HK in blood being only 0.64 μg/ml and 0.36 μg/ml, respectively, 4 h post-injection. By contrast, DEX-modification significantly enhances the blood circulation time of PHPPMs. The drug concentration in plasma was approximately 6.5 μg/ml 12 h post-injection. These results suggested that 2/1PHPPM was safe and could be applied to treat cancer (Ma et al., 2021).

The long blood circulation of cocktail PPM contributes to its accumulation in tumor tissue through the enhanced permeability and retention (EPR) effect (Borresen et al., 2018). The biodistribution of 2/1PHPPM and free drug combination in Hep-2 tumor-bearing mice was investigated to confirm this process. Twelve hours after the injection, the tumor tissue and other major organs were excised and homogenized for drug detection. As shown in Figure 6B, free PTX and free HK accumulated highly in the kidneys, heart, and liver but not in the tumor tissue. 2/1PHPPM showed more drug accumulation at the tumor site than free drug groups. The concentration of 2/1PHPPM in tumor tissue was 3.82- and 4.31-fold higher than that of free PTX and HK, respectively. These results showed that the cocktail PPMs could enrich tumors via the EPR effect.

### Synergistic Antitumor Effects of Cocktail Polymeric Prodrug Micelle *in Vivo*

**FIGURE 7:**
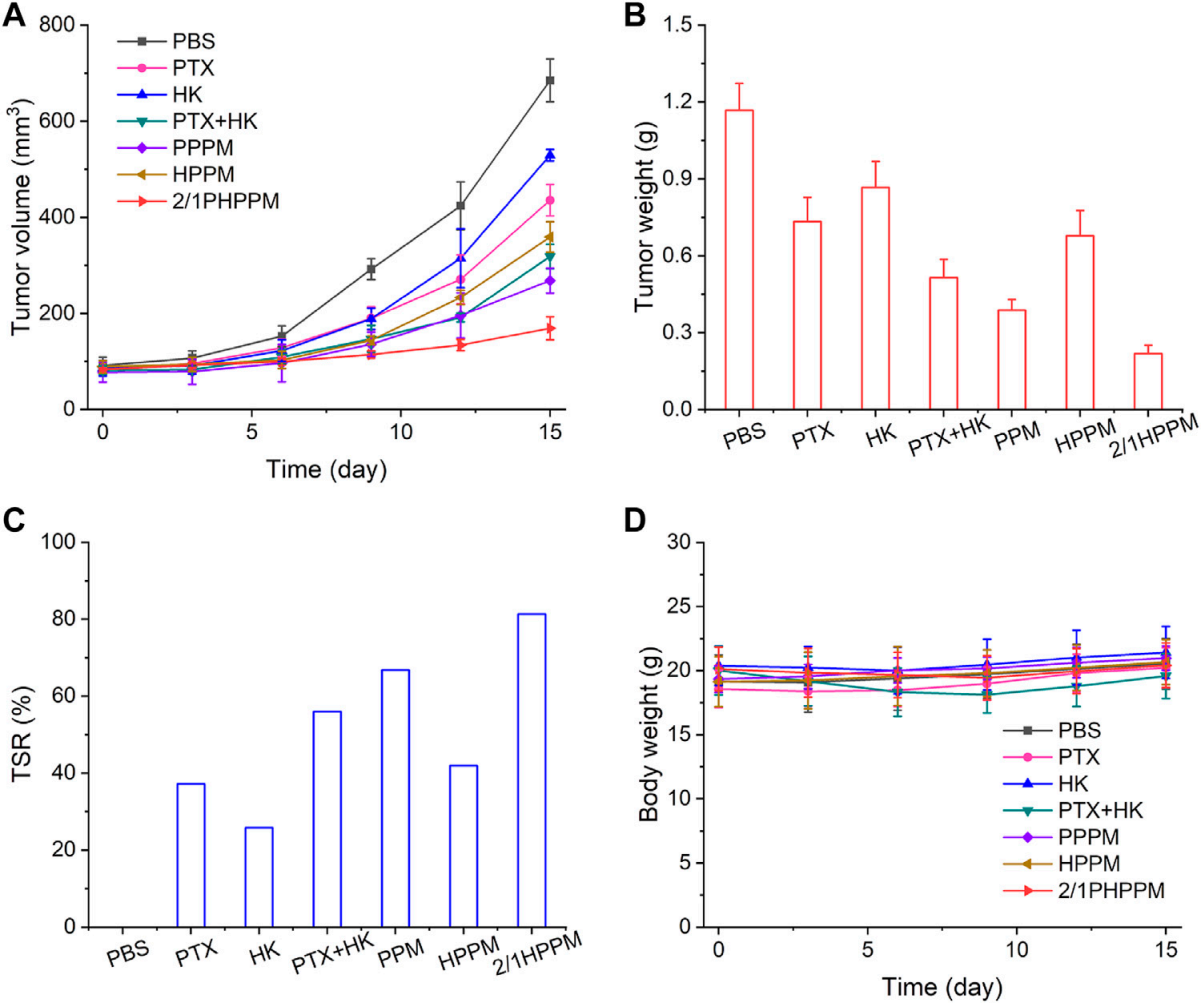
*In vivo* tumor suppression efficiency of different drug formulations. **(A–C)** time-dependent tumor volume changes **(A)**, tumor weight **(B)**, tumor suppression rate at day 15 **(C)**, and time-dependent mice body weight changes **(D)** in mice from different groups over 15 days (*n* = 5). Data are represented as mean ± standard deviation.

Considering the excellent synergistic cancer cell inhibitory effects, high bioavailability, and optimal tumor tissue accumulation of the cocktail PPM, the antitumor efficiency of 2/1PHPPM was further evaluated *in vivo* in Hep-2 tumor-bearing mice. When the tumor volume reached approximately 100 mm^3^, the mice were divided into seven groups (*n* = 5): PBS, PTX, HK, PTX + HK, 2/1PHPPM, PPPM, and HPPM. Tumor volume change curves, tumor weights at day 15, and TSR are presented in Figures 7A–C. On day 15, the tumor volume and weight in the PBS group were approximately 685 mm^3^ and 1.2 g, respectively. When compared to the PBS group, free PTX and HK showed a weak tumor inhibition effect with a TSR of 37.2% and 25.8%, respectively. Although the *in vitro* anticancer efficacy of the PTX + HK group was higher than that of PTX and HK alone, the *in vivo* tumor suppression efficacy of the PTX + HK group was not significantly different than that of monotherapy. This is because the pharmacokinetics of the two drugs are incompatible, preventing them from reaching tumor tissue in the prescribed proportions. As expected, 2/1PHPPM groups showed the best tumor inhibitory effect, with a TSR of 81.3%, compared to the other groups. This was because of tumor accumulation in a precise combination ratio and synergistic antitumor effects of PTX and HK. Moreover, there was no remarkable change in the body weight of mice, indicating that the developed 2/PHPPM had little systematic toxicity (Figure 7D).

## Conclusion

A GSH/ROS dual responsive cocktail PPM co-assembled by PTX and HK polymeric prodrug for suppressing the growth of laryngeal carcinoma was successfully prepared in this study. Both drugs can be loaded ratiometrically in the cocktail PPM, delivered to tumor tissue for quantitative combination, and synchronized drug release intracellular *in vivo*. These advantages endowing the cocktail PPM effectively inhibited tumor growth *in vitro* and *in vivo*. The redox-responsive cocktail PPM with precise drug loading may have a high clinical potential for treating laryngeal carcinoma.

## Data Availability

The original contributions presented in the study are included in the article/Supplementary Material. Further inquiries can be directed to the corresponding author.

## References

[B1] Banerjee V. Sharda N. Huse J. Singh D. Sokolov D. Czinn S. J. (2021). Synergistic Potential of Dual Andrographolide and Melatonin Targeting of Metastatic Colon Cancer Cells: Using the Chou-Talalay Combination Index Method. Eur. J. Pharmacol. 897, 173919. 10.1016/j.ejphar.2021.173919 33577837

[B2] Banik K. Ranaware A. M. Deshpande V. Nalawade S. P. Padmavathi G. Bordoloi D. (2019). Honokiol for Cancer Therapeutics: A Traditional Medicine that Can Modulate Multiple Oncogenic Targets. Pharmacol. Res. 144, 192–209. 10.1016/j.phrs.2019.04.004 31002949

[B3] Bao Y. Guo Y. Zhuang X. Li D. Cheng B. Tan S. (2014). D-α-Tocopherol Polyethylene Glycol Succinate-Based Redox-Sensitive Paclitaxel Prodrug for Overcoming Multidrug Resistance in Cancer Cells. Mol. Pharm. 11 (9), 3196–3209. 10.1021/mp500384d 25102234

[B4] Bonomi M. R. Blakaj A. Blakaj D. (2018). Organ Preservation for Advanced Larynx Cancer: A Review of Chemotherapy and Radiation Combination Strategies. Oral Oncol. 86, 301–306. 10.1016/j.oraloncology.2018.10.004 30409316

[B5] Børresen B. Henriksen J. R. Clergeaud G. Jørgensen J. S. Melander F. Elema D. R. (2018). Theranostic Imaging May Vaccinate against the Therapeutic Benefit of Long Circulating PEGylated Liposomes and Change Cargo Pharmacokinetics. ACS Nano 12 (11), 11386–11398. 10.1021/acsnano.8b06266 30372038

[B6] Cai Y. Xu Z. Shuai Q. Zhu F. Xu J. Gao X. (2020). Tumor-targeting Peptide Functionalized PEG-PLA Micelles for Efficient Drug Delivery. Biomater. Sci. 8 (8), 2274–2282. 10.1039/c9bm02036e 32162618

[B7] Chen F. Huang G. Huang H. (2020). Preparation and Application of Dextran and its Derivatives as Carriers. Int. J. Biol. Macromol. 145, 827–834. 10.1016/j.ijbiomac.2019.11.151 31756474

[B8] Chou T. C. (2010). Drug Combination Studies and Their Synergy Quantification Using the Chou-Talalay Method. Cancer Res. 70 (2), 440–446. 10.1158/0008-5472.CAN-09-1947 20068163

[B9] Chou T. C. (2018). The Combination Index (CI < 1) as the Definition of Synergism and of Synergy Claims. Synergy 7, 49–50. 10.1016/j.synres.2018.04.001

[B10] Dasari S. Njiki S. Mbemi A. Yedjou C. G. Tchounwou P. B. (2022). Pharmacological Effects of Cisplatin Combination with Natural Products in Cancer Chemotherapy. Int. J. Mol. Sci. 23 (3), 1532. 10.3390/ijms23031532 35163459 PMC8835907

[B11] Dikalov S. Losik T. Arbiser J. L. (2008). Honokiol Is a Potent Scavenger of Superoxide and Peroxyl Radicals. Biochem. Pharmacol. 76 (5), 589–596. 10.1016/j.bcp.2008.06.012 18640101 PMC2575413

[B12] Fu B. Wang N. Tan H. Y. Li S. Cheung F. Feng Y. (2018). Multi-Component Herbal Products in the Prevention and Treatment of Chemotherapy-Associated Toxicity and Side Effects: A Review on Experimental and Clinical Evidences. Front. Pharmacol. 9, 1394. 10.3389/fphar.2018.01394 30555327 PMC6281965

[B13] Fu D. Ni Z. Wu K. Cheng P. Ji X. Li G. (2021). A Novel Redox-Responsive Ursolic Acid Polymeric Prodrug Delivery System for Osteosarcoma Therapy. Drug Deliv. 28 (1), 195–205. 10.1080/10717544.2020.1870583 33438472 PMC7808744

[B14] Hu C. Zhuang W. Yu T. Chen L. Liang Z. Li G. (2020). Multi-Stimuli Responsive Polymeric Prodrug Micelles for Combined Chemotherapy and Photodynamic Therapy. J. Mater Chem. B 8 (24), 5267–5279. 10.1039/d0tb00539h 32441291

[B15] Huang D. Zhuang Y. Shen H. Yang F. Wang X. Wu D. (2018). Acetal-linked PEGylated Paclitaxel Prodrugs Forming Free-Paclitaxel-Loaded pH-Responsive Micelles with High Drug Loading Capacity and Improved Drug Delivery. Mater Sci. Eng. C Mater Biol. Appl. 82, 60–68. 10.1016/j.msec.2017.08.063 29025675

[B16] Ibrahim A. Twizeyimana E. Lu N. Ke W. Mukerabigwi J. F. Mohammed F. (2019). Reduction-Responsive Polymer Prodrug Micelles with Enhanced Endosomal Escape Capability for Efficient Intracellular Translocation and Drug Release. ACS Appl. Bio Mater 2 (11), 5099–5109. 10.1021/acsabm.9b00769 35021452

[B17] Kumar P. S. Jeyalatha M. V. Malathi J. Ignacimuthu S. (2018). Anticancer Effects of One-Pot Synthesized Biogenic Gold Nanoparticles (Mc-AuNps) against Laryngeal Carcinoma. J. Drug Deliv. Sci. Technol. 44, 118–128. 10.1016/j.jddst.2017.12.008

[B18] Li D. Han J. Ding J. Chen L. Chen X. (2017). Acid-sensitive Dextran Prodrug: A Higher Molecular Weight Makes a Better Efficacy. Carbohydr. Polym. 161, 33–41. 10.1016/j.carbpol.2016.12.070 28189244

[B19] Li T. Xu H. (2020). Selenium-Containing Nanomaterials for Cancer Treatment. Cell Rep. Phys. Sci. 1 (7), 100111. 10.1016/j.xcrp.2020.100111

[B20] Li X. He Y. Hou J. Yang G. Zhou S. (2020). A Time-Programmed Release of Dual Drugs from an Implantable Trilayer Structured Fiber Device for Synergistic Treatment of Breast Cancer. Small 16 (9), e1902262. 10.1002/smll.201902262 31322830

[B21] Lin Y. R. Chen H. H. Ko C. H. Chan M. H. (2007). Effects of Honokiol and Magnolol on Acute and Inflammatory Pain Models in Mice. Life Sci. 81 (13), 1071–1078. 10.1016/j.lfs.2007.08.014 17826802

[B22] Liu T. Xu L. He L. Zhao J. Zhang Z. Chen Q. (2020). Selenium Nanoparticles Regulates Selenoprotein to Boost Cytokine-Induced Killer Cells-Based Cancer Immunotherapy. Nano Today 35, 100975. 10.1016/j.nantod.2020.100975

[B23] Ma X. Zhang T. Qiu W. Liang M. Gao Y. Xue P. (2021). Bioresponsive Prodrug Nanogel-Based Polycondensate Strategy Deepens Tumor Penetration and Potentiates Oxidative Stress. Chem. Eng. J. 420, 127657. 10.1016/j.cej.2020.127657

[B24] Meng Q. Hu H. Jing X. Sun Y. Zhou L. Zhu Y. (2021). A Modular ROS-Responsive Platform Co-Delivered by 10-hydroxycamptothecin and Dexamethasone for Cancer Treatment. J. Control Release 340, 102–113. 10.1016/j.jconrel.2021.10.027 34718005

[B25] Mollazadeh S. Mackiewicz M. Yazdimamaghani M. (2021). Recent Advances in the Redox-Responsive Drug Delivery Nanoplatforms: A Chemical Structure and Physical Property Perspective. Mater Sci. Eng. C Mater Biol. Appl. 118, 111536. 10.1016/j.msec.2020.111536 33255089 PMC13309921

[B26] Singh N. Sahoo S. K. Kumar R. (2020). Hemolysis Tendency of Anticancer Nanoparticles Changes with Type of Blood Group Antigen: An Insight into Blood Nanoparticle Interactions. Mater Sci. Eng. C Mater Biol. Appl. 109, 110645. 10.1016/j.msec.2020.110645 32228982

[B27] Su X. Ma B. Hu J. Yu T. Zhuang W. Yang L. (2018). Dual-Responsive Doxorubicin-Conjugated Polymeric Micelles with Aggregation-Induced Emission Active Bioimaging and Charge Conversion for Cancer Therapy. Bioconjug Chem. 29 (12), 4050–4061. 10.1021/acs.bioconjchem.8b00671 30404436

[B28] Sui B. Cheng C. Wang M. Hopkins E. Xu P. (2019). Heterotargeted Nanococktail with Traceless Linkers for Eradicating Cancer. Adv. Funct. Mater 29, 1906433. 10.1002/adfm.201906433 33041742 PMC7546548

[B29] Sun B. Luo C. Zhang X. Guo M. Sun M. Yu H. (2019). Probing the Impact of Sulfur/Selenium/Carbon Linkages on Prodrug Nanoassemblies for Cancer Therapy. Nat. Commun. 10 (1), 3211. 10.1038/s41467-019-11193-x 31324811 PMC6642185

[B30] Sung H. Ferlay J. Siegel R. L. Laversanne M. Soerjomataram I. Jemal A. (2021). Global Cancer Statistics 2020: GLOBOCAN Estimates of Incidence and Mortality Worldwide for 36 Cancers in 185 Countries. CA Cancer J. Clin. 71 (3), 209–249. 10.3322/caac.21660 33538338

[B31] Tabatabaei Rezaei S. J. Sarbaz L. Niknejad H. (2016). Folate-Decorated Redox/pH Dual-Responsive Degradable Prodrug Micelles for Tumor Triggered Targeted Drug Delivery. RSC Adv. 6 (67), 62630–62639. 10.1039/c6ra11824k

[B32] Taresco V. Alexander C. Singh N. Pearce A. K. (2018). Stimuli-Responsive Prodrug Chemistries for Drug Delivery. Adv. Ther. 1 (4), 1800030. 10.1002/adtp.201800030

[B33] Tong X. Shi Z. Xu L. Lin J. Zhang D. Wang K. (2020). Degradation Behavior, Cytotoxicity, Hemolysis, and Antibacterial Properties of Electro-Deposited Zn-Cu Metal Foams as Potential Biodegradable Bone Implants. Acta Biomater. 102, 481–492. 10.1016/j.actbio.2019.11.031 31740321

[B34] Tsai H. M. (2019). Atypical Hemolytic Uremic Syndrome: Beyond Hemolysis and Uremia. Am. J. Med. 132 (2), 161–167. 10.1016/j.amjmed.2018.08.011 30145224

[B35] Tu X. He L. Huang H. Ye H. Sun L. Yi L. (2021). Thiolysis of CBD Arylethers for Development of Highly GSH-Selective Fluorescent Probes. Chem. Commun. (Camb) 57 (70), 8802–8805. 10.1039/d1cc03893a 34382627

[B36] Wang D. Zhou N. Zhang N. Zhi Z. Shao Y. Meng L. (2021). Facile Preparation of pH/Redox Dual-Responsive Biodegradable Polyphosphazene Prodrugs for Effective Cancer Chemotherapy. Colloids Surf. B Biointerfaces 200, 111573. 10.1016/j.colsurfb.2021.111573 33476954

[B37] Wang Y. Zhang Z. Zheng C. Zhao X. Zheng Y. Liu Q. (2021). Multistage Adaptive Nanoparticle Overcomes Biological Barriers for Effective Chemotherapy. Small 17 (31), 2100578. 10.1002/smll.202100578 34190401

[B38] Wang N. Wang Z. Nie S. Song L. He T. Yang S. (2017). Biodegradable Polymeric Micelles Coencapsulating Paclitaxel and Honokiol: a Strategy for Breast Cancer Therapy *In Vitro* and *In Vivo* . Int. J. Nanomedicine 12, 1499–1514. 10.2147/IJN.S124843 28260895 PMC5328141

[B39] Wei D. Yu Y. Zhang X. Wang Y. Chen H. Zhao Y. (2020). Breaking the Intracellular Redox Balance with Diselenium Nanoparticles for Maximizing Chemotherapy Efficacy on Patient-Derived Xenograft Models. ACS Nano 14, 16984–16996. 10.1021/acsnano.0c06190 33283501

[B40] Wu D. Zhao Z. Wang N. Zhang X. Yan H. Chen X. (2020). Fluorescence Imaging-Guided Multifunctional Liposomes for Tumor-specific Phototherapy for Laryngeal Carcinoma. Biomater. Sci. 8 (12), 3443–3453. 10.1039/d0bm00249f 32412569

[B41] Xu Q. Yi L. T. Pan Y. Wang X. Li Y. C. Li J. M. (2008). Antidepressant-like Effects of the Mixture of Honokiol and Magnolol from the Barks of Magnolia Officinalis in Stressed Rodents. Prog. Neuropsychopharmacol. Biol. Psychiatry 32 (3), 715–725. 10.1016/j.pnpbp.2007.11.020 18093712

[B42] Zeinali M. Abbaspour-Ravasjani S. Ghorbani M. Babazadeh A. Soltanfam T. Santos A. C. (2020). Nanovehicles for Co-delivery of Anticancer Agents. Drug Discov. Today 25 (8), 1416–1430. 10.1016/j.drudis.2020.06.027 32622880

[B43] Zhang P. Zhang Y. Ding X. Shen W. Li M. Wagner E. (2020). A Multistage Cooperative Nanoplatform Enables Intracellular Co-delivery of Proteins and Chemotherapeutics for Cancer Therapy. Adv. Mater 32 (46), e2000013. 10.1002/adma.202000013 33035385

[B44] Zhang R. X. Wong H. L. Xue H. Y. Eoh J. Y. Wu X. Y. (2016). Nanomedicine of Synergistic Drug Combinations for Cancer Therapy - Strategies and Perspectives. J. Control Release 240, 489–503. 10.1016/j.jconrel.2016.06.012 27287891 PMC5064882

[B45] Zhang Z. Yu M. An T. Yang J. Zou M. Zhai Y. (2019). Tumor Microenvironment Stimuli-Responsive Polymeric Prodrug Micelles for Improved Cancer Therapy. Pharm. Res. 37 (1), 4. 10.1007/s11095-019-2709-1 31823030

[B46] Zhuang W. Xu Y. Li G. Hu J. Ma B. Yu T. (2018). Redox and pH Dual-Responsive Polymeric Micelles with Aggregation-Induced Emission Feature for Cellular Imaging and Chemotherapy. ACS Appl. Mater Interfaces 10 (22), 18489–18498. 10.1021/acsami.8b02890 29737837

[B47] Zou Y. Zhou Y. Jin Y. He C. Deng Y. Han S. (2018). Synergistically Enhanced Antimetastasis Effects by Honokiol-Loaded pH-Sensitive Polymer-Doxorubicin Conjugate Micelles. ACS Appl. Mater Interfaces 10 (22), 18585–18600. 10.1021/acsami.8b04854 29749228

[B48] Zuo S. Sun B. Yang Y. Zhou S. Zhang Y. Guo M. (2020). Probing the Superiority of Diselenium Bond on Docetaxel Dimeric Prodrug Nanoassemblies: Small Roles Taking Big Responsibilities. Small 16 (45), e2005039. 10.1002/smll.202005039 33078579

