# Supplementary material for: Oxidation and Reduction Dual-Responsive Polymeric Prodrug Micelles Co-delivery Precisely Prescribed Paclitaxel and Honokiol for Laryngeal Carcinoma Combination Therapy
Source: Front Pharmacol. 2022 Jul 22;13:934632. doi: 10.3389/fphar.2022.934632 (PMC9354237; doi:10.3389/fphar.2022.934632)
Supplement: Supplementary file 1 [file DataSheet1.docx]

*Supporting Information*

**Oxidation and reduction dual-responsive polymeric prodrug micelles co-delivery precisely prescribed paclitaxel and honokiol for laryngeal carcinoma combination therapy**

*Lanzhu Zhou^#^, Jun Wu^#^, Zhe Sun, Wenzhong Wang*

Department of Otorhinolaryngology Head and Neck Surgery, the First Affiliated Hospital of Bengbu Medical College, Bengbu, 233004, China.

**Corresponding Author:**

Wenzhong Wang,

**Address:** Department of Otorhinolaryngology Head and Neck Surgery, the First Affiliated Hospital of Bengbu Medical College, Bengbu, 233004, China.

**Email:** 13955259093@163.com

**^#^Zhou L and Wu J contributed equally to this work.**

**Table of Contents:**

Supplementary materials

**S1.** Chemicals and reagents

**S2.** Instruments

**S3.** Cell culture

**S4.** Animals

**S5.** Critical micelle concentration detection

Supplementary figures

**Fig. S1** The synthesis routes of DEX-SeSe-PTX and DEX-SeSe-HK.

**Fig. S2** The standard curves for HPLC of PTX and HK.

**Fig. S3** The critical micelle concentrations of DEX-SeSe-PTX and DEX-SeSe-HK.

**Fig. S4** Stability evaluation.

**Fig. S5** Hemolysis rate.

Supplementary tables

**Table S1.** The physicochemical characteristics of PPMs.

**Supplementary Materials**

**S1. Chemicals and reagents**

3,3′-diselenodipropionicacid (DSPA) was purchased from Shanghai Naford Biological company, China. HK, PTX, 4-dimethylaminopyridine (DMAP), and *N,N'*-carbonyldiimidazole (CDI) were purchased from Macklin Reagent (Shanghai, China). DEX (Molecular weight: 10.0 kDa) was obtained from Shanghai Yuanye Bio-Technology company (Shanghai, China). MTT assay kit and DAPI staining solution were purchased from Beyotime Biotechnology Co., Ltd (Shanghai, China).

**S2. Instruments**

Nuclear magnetic resonance spectroscopy (^1^H NMR, Bruker Avance 500 spectrometer) was used to confirm the chemical structure of prodrugs. The particle morphology was examined using a transmission electron microscope (TEM, JEM-2100, JEOL, Japan), and the particle size and zeta potential were detected by the dynamic light scattering (DLS) technique on a Nano-ZS 90 Zetasizer (Malvern, UK).

The drug loading capability of PTX and HK was measured by high-performance liquid chromatography (HPLC) on a SHIMADU LC-20 system (SHIMADU, Japan). For PTX detection water and acetonitrile (35: 65, v/v) were selected as the elution, and the UV detector wavelength was set to 227 nm. For HK detection, the composition of the mobile phase was water, methanol, and acetonitrile (25: 55: 20, v/v/v), and the UV detection wavelength was 294 nm.

**S3. Cell culture**

The laryngeal carcinoma cell line Hep-2 was cultured in Roswell Park memorial institute medium (RPMI) 160 medium supplemented with 10% FBS, 100 U/mL penicillin, and 100 mg/mL streptomycin in an incubator at 37°C and 5% carbon dioxide atmosphere.

**S4. Animals**

Sprague-Dawley (SD) rats (male, 350–450 g, 5–6 weeks) and BALB/c nude mice (male, 16–22 g, 5–6 weeks) were obtained from the Laboratory Animal Center of Bengbu Medical College. All the animals were fed in the Experimental Animal Center of Bengbu Medical College under pathogen-free conditions. All *in vivo* studies were approved by the Institutional Animal Care and Use Committees of the Bengbu Medical College.

**S5. Critical micelle concentration (CMC) detection**

A stock solution of polymeric prodrugs (4 mg/mL) was prepared in PBS with sonication. A known amount of Nile Red in CH_2_Cl_2_ was added to a series of vials and was then evaporated. Subsequently, a measured amount of stock solution was added to each vial, followed by PBS to increase the concentration from 0.01 µg/mL to 600 µg /mL. The final concentration of Nile Red was fixed at 1 ×10^-6^ M. The vials were stirred overnight at room temperature to equilibrate the Nile Red with the micelles and their fluorescence intensity was then recorded. Using a graph of fluorescence intensity vs. log (polymeric prodrug concentration), the CMC was determined as the intersection of the tangents to the two linear portions of the graph.

**Supplementary Figures**





**Fig. S1** The synthesis routes of DEX-SeSe-PTX and DEX-SeSe-HK.


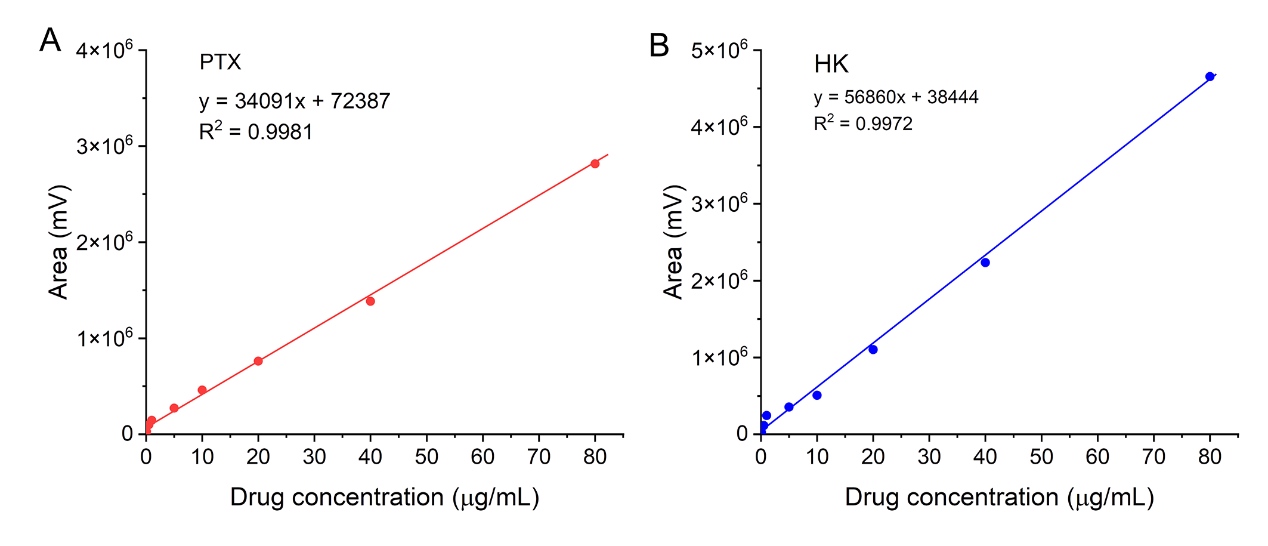


**Fig. S2** The standard curves for HPLC of PTX and HK.


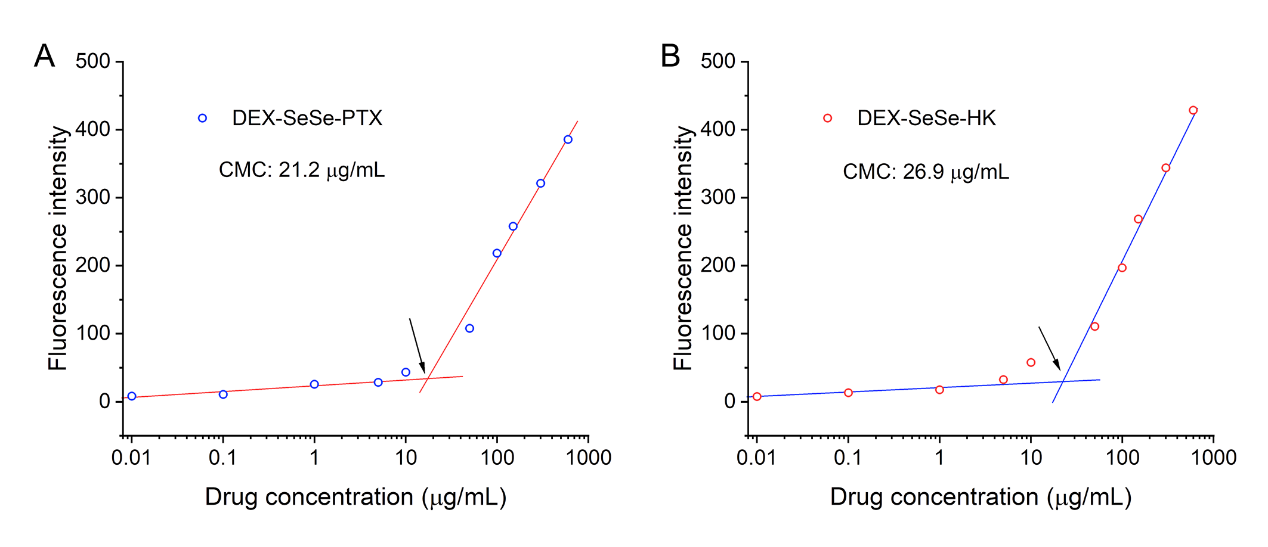


**Fig. S3** The critical micelle concentrations of DEX-SeSe-PTX and DEX-SeSe-HK.


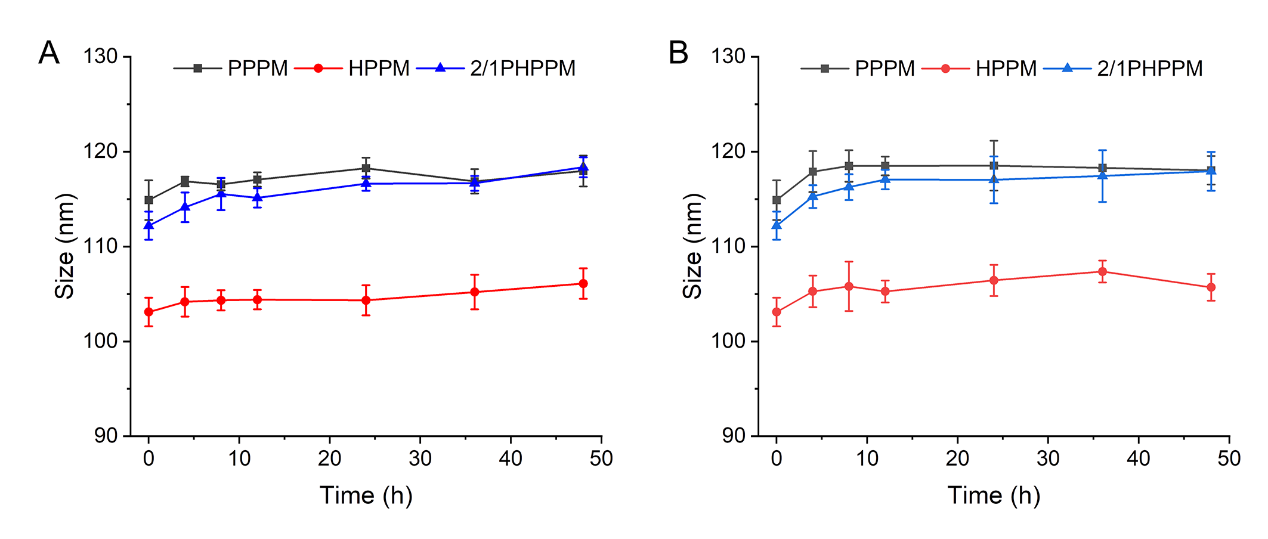


**Fig. S4** Stability evaluation. Size changes of PPPM, HPPM, and 2/1PHPPM in phosphate-buffered saline (pH 7.4) with (A) or without (B) 10% fetal bovine serum. Data showed as mean ± standard deviation, *n* = 3.


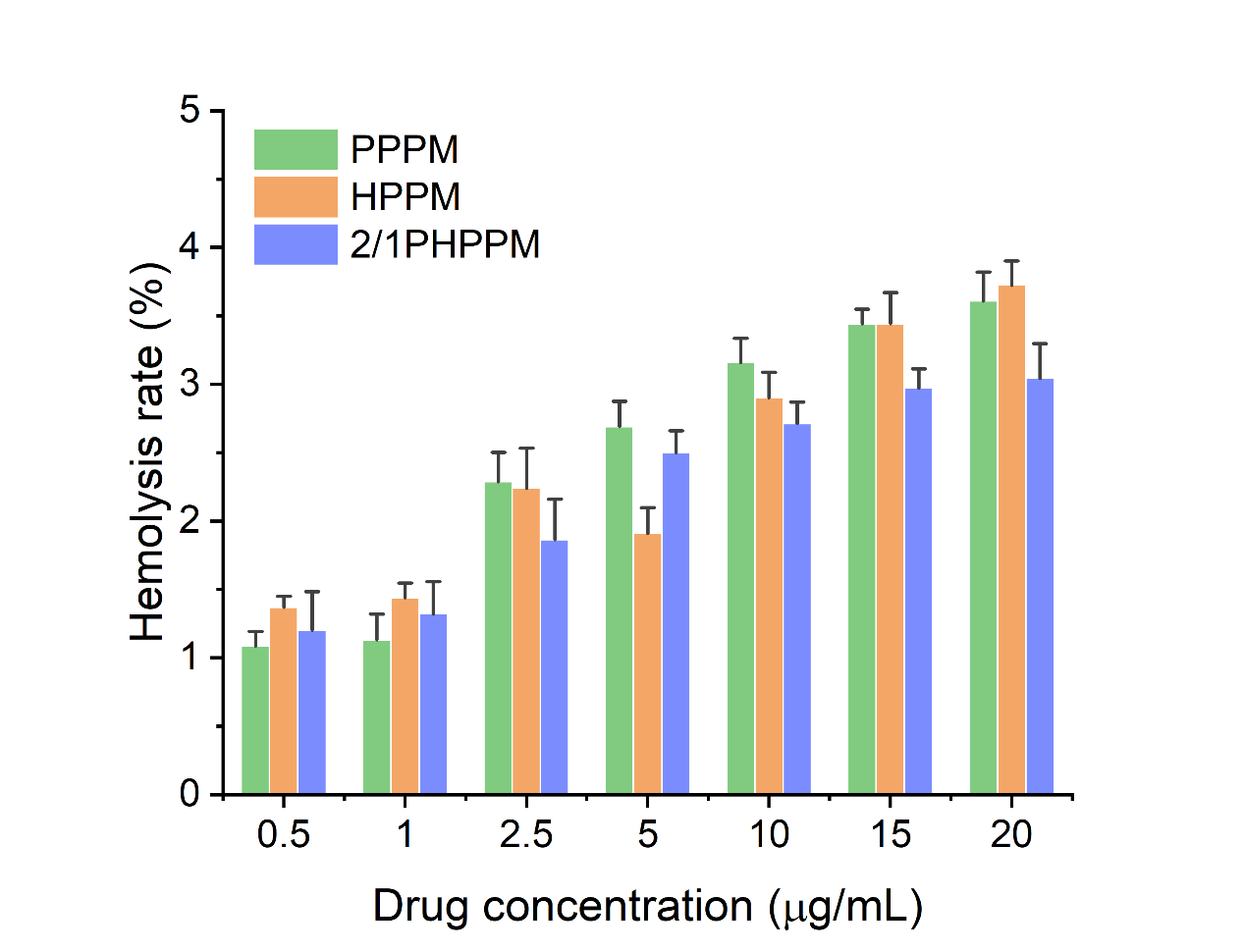


**Fig. S5** Hemolysis rate.

**Supplementary Tables**

**Table S1.** The physicochemical characteristics of PPMs (*n* = 3).

| PPMs | DLC of PTX (%) | DLC of HK (%) | PTX/HK ratio |
| --- | --- | --- | --- |
| PPPM | 4.78 ± 0.35 | - | - |
| HPPM | - | 7.58 ± 0.08 | - |
| 2/5PHPPM | 2.01 ± 0.21 | 5.01 ± 0.26 | 2.01/5.01 |
| 2/3PHPPM | 2.01 ± 0.26 | 2.96 ± 0.42 | 2.01/2.96 |
| 2/1PHPPM | 1.99 ± 0.16 | 1.01 ± 0.64 | 1.99/1.01 |
| 1/1PHPPM | 2.04 ± 0.31 | 2.05 ± 0.41 | 2.04/2.05 |
| 1/2PHPPM | 1.06 ± 0.0.12 | 1.99 ± 0.12 | 1.06/1.99 |
| 1/3PHPPM | 1.00 ± 0.18 | 2.06 ± 0.42 | 1.00/2.06 |
